# The NEI/NCBI dbGAP database: Genotypes and haplotypes that may specifically predispose to risk of neovascular age-related macular degeneration

**DOI:** 10.1186/1471-2350-9-51

**Published:** 2008-06-09

**Authors:** Hong Zhang, Margaux A Morrison, Andy DeWan, Scott Adams, Michael Andreoli, Nancy Huynh, Maureen Regan, Alison Brown, Joan W Miller, Ivana K Kim, Josephine Hoh, Margaret M DeAngelis

**Affiliations:** 1 Department of Ophthalmology, Harvard Medical School, Massachusetts Eye and Ear Infirmary, Boston, MA, USA; 2 Department of Epidemiology and Public Health, Yale University School of Medicine, New Haven, CT, USA; 3 Partner's Healthcare Center for Genetics and Genomics, Harvard Medical School, Cambridge, MA, USA

## Abstract

**Background:**

To examine if the significantly associated SNPs derived from the genome wide allelic association study on the AREDS cohort at the NEI (dbGAP) specifically confer risk for neovascular age-related macular degeneration (AMD). We ascertained 134 unrelated patients with AMD who had one sibling with an AREDS classification 1 or less and was past the age at which the affected sibling was diagnosed (268 subjects). Genotyping was performed by both direct sequencing and Sequenom iPLEX system technology. Single SNP analyses were conducted with McNemar's Test (both 2 × 2 and 3 × 3 tests) and likelihood ratio tests (LRT). Conditional logistic regression was used to determine significant gene-gene interactions. LRT was used to determine the best fit for each genotypic model tested (additive, dominant or recessive).

**Results:**

Before release of individual data, *p*-value information was obtained directly from the AREDS dbGAP website. Of the 35 variants with *P *< 10^-6 ^examined, 23 significantly modified risk of neovascular AMD. Many variants located in tandem on 1q32-q22 including those in *CFH*, *CFHR4*, *CFHR2*, *CFHR5*, *F13B*, *ASPM *and *ZBTB *were significantly associated with AMD risk. Of these variants, single SNP analysis revealed that *CFH *rs572515 was the most significantly associated with AMD risk (P < 10^-6^). Haplotype analysis supported our findings of single SNP association, demonstrating that the most significant haplotype, GATAGTTCTC, spanning *CFH*, *CFHR4*, and *CFHR2 *was associated with the greatest risk of developing neovascular AMD (*P *< 10^-6^). Other than variants on 1q32-q22, only two SNPs, rs9288410 (*MAP2*) on 2q34-q35 and rs2014307 (*PLEKHA1*/*HTRA1*) on 10q26 were significantly associated with AMD status (*P *= .03 and *P *< 10^-6 ^respectively). After controlling for smoking history, gender and age, the most significant gene-gene interaction appears to be between rs10801575 (*CFH*) and rs2014307 (*PLEKHA1*/*HTRA1*) (*P *< 10^-11^). The best genotypic fit for rs10801575 and rs2014307 was an additive model based on LRT. After applying a Bonferonni correction, no other significant interactions were identified between any other SNPs.

**Conclusion:**

This is the first replication study on the NEI dbGAP SNPs, demonstrating that alleles on 1q, 2q and 10q may predispose an individual to AMD.

## Background

Advanced age-related macular degeneration (AMD) is the most common cause of legal blindness in developed countries. Clinically two forms of advanced AMD are recognized: geographic atrophy and neovascular. Geographic atrophy is characterized by a slow progressive degeneration of the retinal pigment epithelium (RPE), resulting in the gradual loss of the photoreceptors. The neovascular form is characterized by the growth of new abnormal blood vessels from beneath the retina that can cause severe and rapid vision loss due to hemorrhage and exudation. It is the neovascular form that is responsible for the majority of debilitating vision loss due to AMD, greatly impairing an individual's ability to read, drive and recognize faces. The burden on public health is significant, as in the U.S. alone, it is predicted that about 3 million individuals over the age of 50 years will have advanced AMD in at least one eye by 2020 [1].

Although the newest treatments for neovascular AMD offer some chance of visual improvement, they require invasive delivery methods, cannot prevent disease, and have limited ability to reverse vision loss. Assessments of an individual's risk of developing advanced AMD are based on ocular findings in those who already have the early stages. Methods are needed for determining which members of the population are at highest risk of vision loss due to AMD prior to the development of any signs of the disease. The identification of genetic variants that could be used as biomarkers would help to predict risk for more advanced stages of AMD and possibly provide new targets for therapeutic or behavioral intervention, thereby reducing or preventing the incidence of this disease.

In an effort to correlate genotypes with advanced AMD clinical phenotypes, the National Eye Institute (NEI) performed a genome wide association study on the Age-related eye disease study (AREDS) cohort consisting of 395 advanced AMD (both neovascular and geographic atrophy) cases and 198 control subjects. Affymetrix and Illumina 100 K arrays were used to test for individual SNP (Single Nucleotide Polymorphism) associations [2,3]In the study presented here, we sought to validate these findings in samples from unrelated patients with only neovascular AMD who had one sibling with normal maculae and was past the age at which the affected sibling was diagnosed with neovascular AMD. We employed this approach to examine if some or all of the NEI/NCBI dbGAP SNPs specifically predisposed an individual to AMD by studying subjects with and without the neovascular form of AMD. Our phenotypically well-defined cohort of 268 subjects comprised 134 extremely discordant sibpairs; that is, pairs with one member in the upper 10% of disease severity (affected sibling) and the other member in the bottom 10–30% of disease severity (unaffected sibling). Mathematical analyses indicate that the evaluation of sibpairs who are extremely discordant for a quantitative, multifactorial trait can be informative for identifying the genetic variants that govern the trait [4]. Many studies have suggested various ways for quantifying AMD but they are not conclusive. However, we know from studies based on prevalence of both early and late AMD that our unaffected siblings likely represent the bottom 10%–30% of the population and those with the most severe forms of AMD (geographic atrophy or neovascular AMD) represent the top 10% with respect to phenotype [5-10].

Moreover, extremely discordant sibpairs can provide a robust alternative study design for finding an association between a genetic variant and a complex disease to using sibpairs with intermediate phenotypes [11] or case-controls study design [12].

We have previously demonstrated that such types of sibpairs can be powerful in identifying the contribution that many genetic variants, even those with a modest effect, along with smoking make simultaneously to AMD susceptibility [13,14]. We employed a combination of the Sequenom iPLEX system technology and direct sequencing to genotype 35 variants in order to identify the contribution that the dbGAP allelic risk factors make independently, and in combination, along with smoking to overall risk of developing neovascular AMD.

## Results

We identified all 34 variants we sought to investigate in our extremely discordant sibpair cohort of 268 Caucasian subjects. In our analysis we also included *CFH *Y402H which has been previously analyzed in this cohort [13]. No significant deviations from Hardy-Weinberg equilibrium for any of the variants studied were observed in the unaffected siblings, indicating unlikely contamination of our dataset. For genotype and allele frequencies for each of these SNPs, please see Additional File 1.

**Figure 1:**
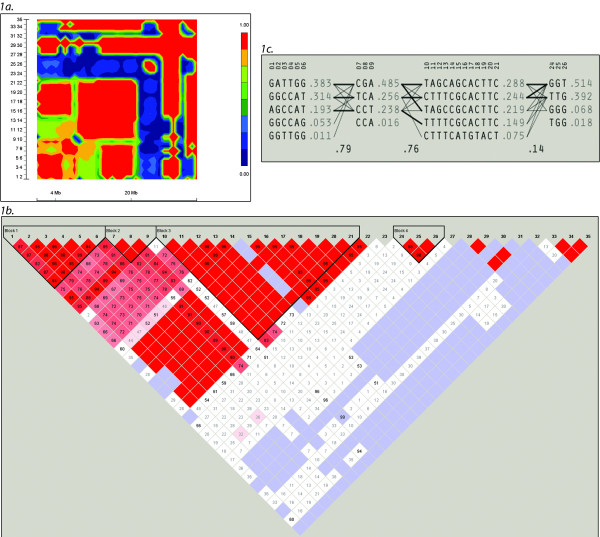
**1a**. **Linkage disequilibrium (*D*') between the genotyped SNPs as shown in a heat map.** SNPs encompassing the1q22-q32 region are represented by 3 distinct haplotype blocks. The more red the color, the higher the LD which is indicated by the gradient bar to the right of the LD plot. **1b**. Linkage disequilibrium (*D*') between the genotyped SNPs as shown in an LD plot. **1c**. Haplotypes Haplotypes of SNPs together with estimated frequencies are shown. The values of multi-allelic D' (.79, .76, and .14) reflect the level of recombination between adjacent blocks. **Abbreviations:** bp, base pairs; SNP, Single Nucleotide Polymorphism; Linkage disequilibrium (*D*^'^).

The results of both types of McNemar's tests, likelihood ratio tests and resulting *P *values for all 35 SNPs after controlling for age, gender and smoking are shown in Table 1. The resulting odds ratios, confidence intervals and mode of inheritance resulting from the LRT also appear in Table 1. In addition to the Y402H *CFH *variant, 23 SNPs were significantly associated with neovascular AMD risk (Table 1). PHASE produced similar results to SNPHAP for diplotype reconstruction so we focused the remainder of our analysis using SNPHAP. Many variants located in tamdem on 1q32-q22 including those in complement Factor H (*CFH*), complement factor H-related 4 (*CFHR4*), complement factor H-related 2 (*CFHR2*), complement factor H-related 5 (*CFHR5*), coagulation factor 13 subunit B (*F13B*), abnormal spindle-like microcephaly associated (*ASPM*) and zinc finger and BTB domain containing 41 homolog (*ZBTB41*) were significantly associated with AMD risk (Table 1). According to LRT, single SNP analysis showed that *CFH *rs572515 on 1q25 was the most strongly associated SNP with AMD risk under an additive model. All SNPs analyzed on 1q32-q22 except for *F13B *rs2990510 were significantly associated with neovascular AMD risk using the LRT (*P *< .01). SNP rs9288410 (*MAP2*) on 2q35-q34 and SNP rs2014307 on 10q26 *PLEKHA1*/*HTRA1 *were also associated with AMD risk (OR: 1.92; 95% C.I.: 0.89, 4.18; *P *= 0.03 and OR: 0.240; 95% C.I.: 0.11, .52; *P *< 10^-4 ^respectively). According to ENSEMBL [15], SNP rs2014307 is equidistant between the end of *PLEKHA1 *and the beginning of *HTRA1*. Single SNP analysis in the 1q32-q22 region was confirmed by demonstrating that the haplotype GATAGTTCTC for alleles spanning *CFH*, *CFHR4*, and *CFHR2 *were associated with the greatest risk of neovascular AMD (*P *< 10^-6^). These 9 SNPs, which included the Y402H *CFH *variant, spanned the 1q31-1q32 region and formed two haplotype blocks (Figure 1a and Figure 1b) that were in linkage disequilibrium (D' = 0.79) (Figure 1c) (see Table 2).

**Table 1:** Single SNP Analysis

SNP	Gene*	Location*	**2 × 2 McNemar's test *p*-value**	**3 × 3 McNemar's test *p*-value**	**Likelihood Ratio Test *p*-value**	Odds Ratio (95% C.I.) of most likely mode of inheritance	Likelihood Ratio Test Model
rs800292	CFH	1q32	0.0005	1.6766E-05	0.0020	0.295(0.126–0.693)	Additive
rs572515	CFH	1q32	1.1422E-06	2.1771E-09	7.0012E-07	4.619(2.288–9.326)	Additive
rs7529589	CFH	1q32	0.0003	1.6852E-05	1.2034E-05	8.403(2.670–26.442)	Recessive
rs1061170	CFH	1q32	0.0002	3.9821E-05	1.9045E-05	7.382(2.495–21.838)	Recessive
rs12038333	CFH	1q32	0.0001	1.7989E-06	8.3182E-06	3.151(1.707–5.819)	Recessive
rs203674	CFH	1q32	5.7447E-06	5.0608E-08	4.3995E-06	3.914(2.020–7.585)	Additive
rs10801575	~CFH	~1q32	0.0304	0.0315	0.0108	0.370(0.174–0.785)	Dominant
rs1853883	CFHR4	1q32	0.0001	6.9750E-06	0.0003	2.687(1.519–4.751)	Additive
rs3790414	CFHR2	1q31-q32.1	0.0018	0.0002	0.0137	0.424(0.206–0.876)	Additive
rs1759016	CFHR5	1q22-q23	0.0092	0.0037	0.0092	0.465(0.262–0.827)	Additive
rs10922152	CFHR5	1q22-q23	0.0007	0.0001	0.0014	0.434(0.258–0.730)	Additive
rs10922153	CFHR5	1q22-q23	0.0002	1.0290E-05	0.0004	0.389(0.224–0.677)	Additive
rs6663083	?	1q31-1q32	0.0003	1.6704E-05	0.0008	0.419(0.248–0.707)	Additive
rs2990510	F13B	1q31-q32.1	0.0438	0.0395	0.1003	2.462(0.801–7.571)	Recessive
rs6003	F13B	1q31-q32.1	0.0153	0.0022	0.0025	0.162(0.041–0.631)	Additive
rs1412632	?	1q31-1q32	0.0153	0.0022	0.0030	0.165(0.042–0.645)	Additive
rs1412631	?	1q31-1q32	0.0098	0.0005	0.0013	0.110(0.022–0.557)	Additive
rs12677	ASPM	1q31	0.0098	0.0005	0.0019	0.127(0.026–0.609)	Additive
rs4915337	ASPM	1q31	0.0614	0.0233	0.0067	0.182(0.045–0.740)	Additive
rs1888991	ASPM	1q31	0.0244	0.0051	0.0044	0.175(0.044–0.696)	Additive
rs6677082	ASPM	1q31	0.0244	0.0051	0.0048	0.175(0.044–0.695)	Additive
rs6656448	ZBTB41	1q31.3	0.0218	0.0054	0.0042	0.209(0.063–0.694)	Additive
rs9288410	MAP2	2q34-q35	0.5563	0.3721	0.0294	1.923(0.885–4.179)	Recessive
rs304039	ITPR1	3p26-p25	0.6774	0.7070	0.1035	1.943(0.858–4.396)	Recessive
rs304041	ITPR1	3p26-p25	0.5896	0.4206	0.1035	1.943(0.858–4.396)	Recessive
rs1038639	ITPR1	3p26-p25	1.0000	0.9062	0.4345	0.731(0.332–1.610)	Dominant
rs1447338	?	4q34-4q35	0.5419	0.4856	0.2949	1.350(0.730–2.497)	Additive
rs7090030	ADD3	10q24.2-q24.3	1.0000	1.0000	0.5834	0.485(0.035–6.710)	Additive
rs11194995	ADD3	10q24.2-q24.3	1.0000	1.0000	0.5834	0.485(0.035–6.710)	Additive
rs11194996	ADD3	10q24.2-q24.3	1.0000	1.0000	0.9217	0.881(0.071–10.862)	Additive
rs11195001	ADD3	10q24.2-q24.3	1.0000	1.0000	1.0000	0.485(0.035–6.715)	Additive
rs2014307	PLEKHA1/HTRA1	10q26	6.6872E-06	8.1056E-07	3.7092E-05	0.240(0.111–0.520)	Additive
rs949252	GPR152	11q13.1	0.4795	0.4795	0.1979	2.006(0.115–35.135)	Dominant
rs7124630	TMEM134	11q13.1	0.4795	0.0000	0.3864	1.004E-07(0.000-Inf)	Additive
rs11575221	STAT2	12q13-12q14	1.0000	1.0000	0.4358	2.740(0.204–36.835)	Additive

**Abbreviations: **SNP, single nucleotide polymorphism; C.I., confidence interval.

* Gene and location of each SNP determined using ENSEMBL [15].

**Table 2:** Legend of Figure 1.

ID	rs number	Chromosome	Position (bp)
1	rs800292	1	169751219
2	rs572515	1	169755247
3	rs7529589	1	169767262
4	rs1061170	1	169768220
5	rs12038333	1	169781422
6	rs203674	1	169793601
7	rs10801575	1	169966624
8	rs1853883	1	169995661
9	rs3790414	1	170045770
10	rs1759016	1	170077967
11	rs10922152	1	170088475
12	rs10922153	1	170104084
13	rs6663083	1	170106129
14	rs2990510	1	170146127
15	rs6003	1	170156490
16	rs1412632	1	170162337
17	rs1412631	1	170162706
18	rs12677	1	170178842
19	rs4915337	1	170217004
20	rs1888991	1	170236289
21	rs6677082	1	170238000
22	rs6656448	1	170268765
23	rs9288410	2	204266059
24	rs304039	3	4490512
25	rs304041	3	4491054
26	rs1038639	3	4500754
27	rs1447338	4	178374405
28	rs7090030	10	105592344
29	rs11194995	10	105619802
30	rs11194996	10	105621397
31	rs11195001	10	105626325
32	rs2014307	10	117949887
33	rs949252	11	66975485
34	rs7124630	11	66997187
35	rs11575221	12	56407497

Table 3 shows the results for testing interaction, joint effects and main effects of each significantly associated SNP on chromosome 1 with rs2014307 on chromosome 10. After controlling for smoking, age and gender, the resulting *P *values, odds ratios and 95% C.I. of each SNP, while controlling for the other SNP, are shown. Almost all of the combinations of SNP pairs tested showed no interaction at 0.05 level of significance, except for rs800292/rs2014307 and rs3790414/rs2014307. However, neither of these SNP pair combinations was significant after applying a Bonferroni correction. Therefore, no interaction is found in the joint effect and main effect analyses.

**Table 3:** Pairwise SNP Analysis

SNP	Interaction	Joint effect of SNP and rs2014307	Effect of SNP, controlling for rs2014307	Odds Ratio (95% C.I.)	Effect of rs2014307, controlling for SNP	Odds Ratio (95% C.I.)
rs800292	0.034082688	1.51E-06	0.0005	0.369(0.151–0.899)	0.0193	0.271(0.122–0.603)
rs572515	0.560883637	2.03E-10	0.0003	4.817(2.236–10.379)	3.7602E-06	0.233(0.096–0.562)
rs7529589	0.454600765	7.27E-09	0.0007	7.861(2.283–27.066)	0.0001	0.260(0.112–0.605)
rs1061170	0.597380515	2.22E-08	0.0007	6.831(2.113–22.079)	0.0002	0.258(0.111–0.600)
rs12038333	0.393546389	4.22E-08	0.0006	2.884(1.512–5.503)	0.0004	0.272(0.121–0.609)
rs203674	0.463309999	2.68E-09	0.0007	3.721(1.847–7.496)	0.0001	0.256(0.109–0.603)
rs10801575	0.808299262	1.49E-11	4.0182E-05	0.309(0.135–0.710)	0.0078	0.194(0.079–0.475)
rs1853883	0.760773267	9.31E-08	0.0004	2.448(1.355–4.421)	0.0021	0.263(0.117–0.589)
rs3790414	0.088493626	2.96E-06	0.0001	0.463(0.213–1.004)	0.0411	0.252(0.115–0.554)
rs1759016	0.873846771	9.04E-07	0.0001	0.490(0.266–0.902)	0.0244	0.246(0.111–0.545)
rs10922152	0.876195836	1.15E-07	0.0002	0.484(0.282–0.831)	0.0056	0.245(0.107–0.558)
rs10922153	0.518399094	2.99E-09	0.0006	0.413(0.232–0.736)	0.0052	0.259(0.110–0.606)
rs6663083	0.989583181	6.89E-08	0.0001	0.443(0.258–0.761)	0.0015	0.223(0.095–0.523)
rs2990510	0.487660055	4.62E-06	3.9350E-05	2.894(0.874–9.585)	0.0684	0.222(0.099–0.499)
rs6003	0.454199074	4.15E-07	0.0001	0.151(0.035–0.649)	0.0048	0.222(0.098–0.501)
rs1412632	0.396769175	5.61E-07	0.0001	0.164(0.038–0.697)	0.0066	0.226(0.100–0.509)
rs1412631	0.64561949	2.26E-07	0.0001	0.113(0.021–0.592)	0.0025	0.234(0.104–0.527)
rs12677	0.500827762	5.73E-07	0.0002	0.132(0.025–0.707)	0.0068	0.239(0.108–0.528)
rs4915337	0.313727331	1.02E-06	0.0001	0.176(0.040–0.782)	0.0127	0.239(0.108–0.528)
rs1888991	0.454199074	4.15E-07	0.0001	0.151(0.035–0.649)	0.0048	0.222(0.098–0.501)
rs6677082	0.454199074	4.15E-07	4.8640E-05	0.151(0.035–0.649)	0.0048	0.222(0.098–0.501)
rs6656448	0.707806912	1.10E-06	0.0001	0.224(0.062–0.809)	0.0136	0.236(0.106–0.525)

**Abbreviations: **SNP, Single Nucleotide Polymorphism; C.I., Confidence Interval.

The most significant joint effect is observed between rs10801575 and rs2014307 (*P *< 10^-11^). The LRT demonstrated that *CFH *SNP rs10801575 fits best under a dominant model whereas rs2014307 fits best under an additive model. The main effect for each of these SNPs individually is highly significant (OR: 3.16; 95% C.I.: 3.16, 7.347; *P *< 10^-4^and OR: 0.26; 95% C.I.: 0.13, .55; *P *< 10^-6^, respectively).

## Discussion

Inconsistency in replication of findings among studies could possibly be due to population stratification, disease heterogeneity, and/or diagnostic heterogeneity in phenotype among cases [12]. In the study presented here, using phenotypically well defined extremely discordant sibpairs, we were able to confirm the NEI/NCBI dbGAP findings for chromosomes 1, 2 and 10 in a Caucasian population (mostly derived from the U.S.) employing a different methodological design [2,3]. Moreover, our findings suggest that specific genetic variants may predispose to the neovascular form of AMD, as the dbGAP data included cases with both neovascularization and geographic atrophy. On the other hand, it could be that our use of the Bonferroni correction was overly stringent (given the LD between many of the SNPs) and this could account for the fact that we only observed a portion of the variants as being significantly associated with neovascular AMD risk. However, in both study designs (NEI and the study presented here) these variants were only examined for association with advanced stages of AMD, and it may be that these variants are also associated with the early forms of AMD as well. Further, we were able to demonstrate the risk of AMD conferred by the joint effect of these SNPs while controlling for smoking history, age and gender. The most significant joint effect was between rs10801575 (*CFH*) and rs2014307 (*PLEKHA1/HTRA1*) (*P *< 10^-11^). After controlling for the effect of each SNP with respect to the other, it was evident that this joint effect was due to the significant assocation of SNP rs2014307 with AMD risk, as the *p *value was greater for the *PLEKHA1/HTRA1 *SNP (*P *< 10^-6^) than it was for *CFH *SNP rs10801575, (*P *< 10^-4^). While these findings support the contribution of chromosome 1q22-q32, they also demonstrate that the 10q26 region is likely more stongly associated with neovascular AMD risk [13,16-18]. It may be that the dbGAP variants we analyzed on chromosomes 3p, 4q, 11q and 12q predispose an individual to the geographic atrophic subytpe. Whether or not these variants represent true disease causing variants or are in high LD with the susceptibilily allele remains to be determined. We demonstrated that variants in the complement pathway genes *CFH*, *CFHR2*, *CFHR4 *and *CFHR5 *are significantly associated with AMD risk, however variants in these genes may not be independent factors in the pathophysiology of AMD due to the high LD throughout the 1q25-1q32 region [19-25].

We also demonstrated that significantly associated variants in *F13B*, *ASPM*, *MAP2 *and *ZBTB41 *point to other pathways yet to be studied in AMD etiology. *F13B *is the noncatalytic subunit of coagulation factor 13, which upon activation by thrombin, functions to covalently cross-link fibrin, making a clot mechanically stronger and more resistant to fibrinolysis [26]. *F13B *is composed of 641 amino acids, divided into 10 tandem repeats, which is characteristic of the complement activation system regulatory proteins [27] There is also a structural similarity between *F13B *and *CFH *[28,29]. Both *ASPM *and *MAP2 *play a critical role during neurogenesis. *ASPM *regulates brain growth and mutations in this gene were shown to be associated with microcephaly [30-32]. Further ASPM has been implicated in tumor cell proliferation and growth [33]* MAP2 *stabilizes and interacts with microtubules during the growth, differentiation, and development of neurons [34,35]. Further, *ZBTB41 *proteins are transcription regulators, functioning in a wide range of processes, including development, differentiation, and oncogenesis [36,37]. *ZBTB41 *proteins also play an important role in the hematopoietic system, particularly in T cell development and function [38].

## Conclusion

In summary, this is the first report to confirm association of neovascular AMD risk variants obtained from the NEI/NCBI dbGAP database. Moreover, we show that specific variants may predispose to only the neovascular form of AMD, and in agreement with others, show that the 10q26 region is more strongly associated with neovascular AMD risk than 1q.

## Methods

### Patient population

The protocol was reviewed and approved by the Institutional Review Boards at the Massachusetts Eye & Ear Infirmary (MEEI), Boston, Massachusetts and the William Beaumont Hospital, Royal Oak, Michigan and conforms to the tenets of the Declaration of Helsinki. Eligible patients were enrolled in this study after they gave informed consent either in person, over the phone, or through the mail, before answering questions to a standardized questionnaire and donating 10 to 50 ml of venous blood.

Details of the recruitment of patients and their siblings are described elsewhere [14,39]. In brief, all index patients were aged 50 years or older and had the neovascular form of AMD in at least one eye, defined by subretinal hemorrhage, fibrosis, or fluorescein angiographic presence of neovascularization documented at the time of, or prior to, enrollment in the study. Patients whose only exudative finding was a retinal pigment epithelium detachment were excluded because this finding may not represent definite neovascular AMD and, therefore, the severe phenotype we sought. Patients with signs of pathologic myopia, presumed ocular histoplasmosis syndrome, angioid streaks, choroidal rupture, any hereditary retinal diseases other than AMD, and previous laser treatment due to retinal conditions other than AMD were also excluded.

The unaffected siblings had normal maculae at an age older than that at which the index patient was first diagnosed with neovascular AMD. Normal maculae (defined as the zone centered at the foveola and extending 2 disc diameters, or 3000 microns, in radius) fulfilled the following criteria: 0–5 small drusen (all less than 63 microns in diameter), no pigment abnormalities, no geographic atrophy, and no neovascularization (as defined previously [14,39]; AMD "category 1" or less on the AREDS scale). Disease status of every participant was confirmed by at least two of the investigators by evaluation of fundus photographs or fluorescein angiograms except when one of the investigators directly examined an unaffected sibling during a home visit (n = 4 cases). Subject characteristics of our extremely discordant sibpair population are presented in Table 4.

**Table 4:** Subject Characteristics

Population	Mean Age (years)	Range	Standard Deviation	% Male (n/134)
Affected Siblings	71.28	48.97–86.38	8.26	45.5% (61/134)
Unaffected Siblings	72.77	41.32–90.86	8.97	39.6% (53/134)

Additionally, we administered a standardized questionnaire to all eligible participants in person or over the phone to ascertain smoking exposure, with the age of the index patient at the time of the fundus photographs as our cutoff reference age for smoking exposure for both members in a sibship. In most cases, the diagnosis of AMD was made simultaneously with the diagnosis of neovascular AMD. If a participant ever smoked, we recorded the age when they started smoking, the age when they quit smoking (if they did quit), and the number of packs of cigarettes smoked per day, on average. Based on the responses, the number of pack-years of cigarettes smoked was calculated for each smoker. Participants who smoked less than 100 cigarettes during their lifetime (i.e., less than 1/73 of a pack-year) were categorized as having never smoked. A pack-year was defined as one pack of cigarettes per day for one year, with one pack defined as twenty cigarettes.

### Genotyping Analysis

For both the Sequenom iPLEX system technology and direct sequencing protocols, leukocyte DNA was either purified by using standard phenol-chloroform or DNAzol (Invitrogen Corporation, Carlsbad, California) extraction protocols.

Multiplex PCR assays were designed using Sequenom SpectroDESIGNER software (version 3.0.0.3) by inputting sequence containing the SNP site and 100 bp of flanking sequence on either side of the SNP. Briefly, 10 ng genomic DNA was amplified in a 5 ul reaction containing 1 × HotStar Taq PCR buffer (Qiagen), 1.625 mM MgCl_2_, 500 uM each dNTP, 100 nM each PCR primer, 0.5 U HotStar Taq (Qiagen). The reaction was incubated at 94°C for 15 minutes followed by 45 cycles of 94°C for 20 seconds, 56°C for 30 seconds, 72°C for 1 minute, followed by 3 minutes at 72°C. Excess dNTPs were then removed from the reaction by incubation with 0.3 U shrimp alkaline phosphatase (USB) at 37°C for 40 minutes followed by 5 minutes at 85°C to deactivate the enzyme. Single primer extension over the SNP was carried out in a final concentration of between 0.625 uM and 1.5 uM for each extension primer (depending on the mass of the probe), iPLEX termination mix (Sequenom) and 1.35 U iPLEX enzyme (Sequenom) and cycled using a two-step 200 short cycles program; 94°C for 30 seconds followed by 40 cycles of 94°C for 5 seconds, 5 cycles of 52°C for 5 seconds, and 80°C for 5 seconds, then 72°C for 3 minutes. The reaction was then desalted by addition of 6 mg cation exchange resin followed by mixing and centrifugation to settle the contents of the tube. The extension product was then spotted onto a 384 well spectroCHIP before being flown in the MALDI-TOF mass spectrometer. Data was collected, real time, using SpectroTYPER Analyzer 3.3.0.15, SpectraAQUIRE 3.3.1.1 and SpectroCALLER 3.3.0.14 (Sequenom). Additionally, to ensure data quality, genotypes for each subject were also checked manually.

Six of the dbGAP SNPs (rs11575221, rs949252, rs11194995, rs6656448, rs12677, rs6003) were not amenable to genotyping by the Sequenom iPLEX system technology and thus were directly sequenced. Oligonucleotide primers were selected using the Primer3 program [40] to encompass the entire coding region and flanking intronic sequences except in the case of an intronic SNP (rs6656448) where at least 100 base pairs on either side of the variant was sequenced and analyzed (Table 5).

**Table 5:** PCR Conditions

SNPs	Gene	Location	Variant	Forward primer	Reverse Primer
rs11575221	?	?	A/C	TCTCCAGGCTCCTCAAGCTA	CGCCTACAACTTCGGCTAAC
rs949252	GPR152	Exon 01	T/C	CAGCCACAGCTGAACCCTAC	TCTGACTGGCTGGTTCCTCT
rs11194995	ADD3	Intron 12	C/T	TCAAGAGTGTTTTCTTCCCATTT	ATTAGTCGGGCACGGTGA
rs6656448	ZBTB41	Intron 02	G/A	ATTAACACGCCTCCAACCAC	CAACAGTGTTTGGGCTGAGA
rs12677	ASPM	3' UTR	C/T	GGGAAATGATGTGTTCAGGAG	AGGTGTAATCAGCTATTATTTCCTTT
rs6003	F13B	Exon 10	G/A	TCCAAAATGAAATCGCCAAT	GGTGGGTTGTAGGGATTGAG

**Abbreviations: **PCR, Polymerase Chain Reaction; SNP, Single Nucleotide Polymorphism.

For all amplicons, the polymerase chain reaction (PCR) was used to amplify genomic DNA fragments from 20 ng of leukocyte DNA in a solution of 10× PCR buffer containing 25 mM of MgCl_2_, 0.2 mM each of dATP, dTTP, dGTP, and dCTP, and 0.5 units of Taq DNA polymerase (USB Corporation, Cleveland, OH). 5 M Betaine was added to each PCR (Sigma-Aldrich, St. Louis, MO), except for the reactions containing primers used to analyze rs12677 and rs6003. The temperatures used during the polymerase chain reaction were as follows: 95°C for 5 minutes followed by 35 cycles of 58°C for 30 seconds, 72°C for 30 seconds and 95°C for 30 seconds, with a final annealing at 58°C for 1.5 minutes and extension of 72°C for 5 minutes. For sequencing reactions, PCR products were digested according to manufacturer's protocol with ExoSAP-IT (USB Corporation, Cleveland, OH) then were subjected to a cycle sequencing reaction using the Big Dye Terminator v3.1 Cycle Sequencing kit (Applied Biosystems, Foster City, CA) according to manufacturer's protocol. Products were purified with Performa DTR Ultra 96-well plates (Edge Biosystems, Gaithersburg, MD) in order to remove excess dye terminators. Samples were sequenced on an ABI Prism 3100 DNA sequencer (Applied Biosystems, Foster City, CA). Electropherograms generated from the ABI Prism 3100 were analyzed using the Lasergene DNA and protein analysis software (DNASTAR, Inc., Madison, WI). Electropherograms were read by two independent evaluators without knowledge of the subject's disease status. All patients were sequenced in the forward direction (5' to 3'), unless variants or polymorphisms were identified, in which case confirmation was obtained in some cases by sequencing in the reverse direction.

### Statistical Analyses

Statistical analysis was performed using the R and STATA (v8) packages. The McNemar's test was used to evaluate the effect of each SNP individually on risk of AMD. First the standard 2 × 2 table was constructed, and then a modification of the McNemar's test using a 3 × 3 table, $T_{1}=\frac{{\left(|b-c|+1\right)}^{2}}{b+c}$ was also employed to compare the effect of the three genotypes for each variant simultaneously. A Bonferroni correction was applied to all resulting *P *values that were calculated for each allele of the 35 SNPs that met these criteria. Linkage disequilibrium (*r*^2^) between adjacent SNPs on the same chromosome was evaluated. Both SNPHAP and Phase were used separately in cases and controls to reconstruct the diplotype (haplotype pair) for each sample in each SNP group. The posterior probability for any of the resulting diplotypes was calculated. The diplotype with the largest probability (> 0.80 for all of the samples) was picked up as the designated diplotype. For haplotype analysis, SNPs that fit the following criteria were analyzed: selected SNPs on the same chromosome, adjacent SNPs in statistically significant LD, and minor allele frequencies were similar. Both types of McNemar's tests were then used to determine significant haplotypes. Conditional logistic regression (CLR) was performed to identify factors associated with neovascular AMD. Potential risk factors, SNPs, were evaluated initially one at a time. Any significant allelic factors were then tested for significance while controlling for age, gender and smoking history. For each significant SNP, the minor allele (in unaffected siblings) in both the homozygous and heterozygous states versus the common allele in the homozygous state was examined in the model. Gene-gene interaction was tested first by assuming each locus had two alleles, with three possible genotypes and 9 possible two-locus genotypes. The likelihood ratio test (LRT) was used to test the null hypothesis of no gene-gene interaction (logarithm of odds ratio = 0). Three genotypic models were examined (Additive, Dominant, and Recessive) using the likelihood ratio test.

Genotype and allele frequencies for all SNPs identified as significant were calculated in the affected and separately in unaffected siblings [see Additional File 1]. Deviation from Hardy-Weinberg Equilibrium (HWE) was tested on each SNP in unaffected (control) individuals using the chi square test.

## Competing interests

The authors declare that they have no competing interests.

## Authors' contributions

HZ participated in the design of the study, performed the statistical analysis and helped to draft the manuscript. AD participated in the design of the study and performed the statistical analysis. MAM carried out the molecular genetic studies, participated in the sequence alignment, Sequenom analysis and helped to draft the manuscript. SA, MA, NH, MR, AB carried out the molecular genetic studies, participated in the sequence alignment and Sequenom analysis. JWM, IKK participated in the design of the study, recruited and characterized patients. JH conceived of the study, participated in its design and coordination, performed the statistical analysis and helped to draft the manuscript. MMD conceived of the study, participated in its design and coordination, recruited and characterized patents, carried out the molecular genetic studies, participated in the sequence alignment, Sequenom analysis and helped to draft the manuscript. All authors read and approved the final manuscript.

## Pre-publication history

The pre-publication history for this paper can be accessed here:

http://www.biomedcentral.com/1471-2350/9/51/prepub

## Supplementary Material

Additional file 1 "Genotype and Allele Frequencies", is a word file containing the genotype and allele frequencies of all the SNPs genotyped. Click here for file
