# Supplementary material for: The NEI/NCBI dbGAP database: Genotypes and haplotypes that may specifically predispose to risk of neovascular age-related macular degeneration
Source: BMC Med Genet. 2008 Jun 9;9:51. doi: 10.1186/1471-2350-9-51 (PMC2441616; doi:10.1186/1471-2350-9-51)
Supplement: Additional file 1 — "Genotype and Allele Frequencies", is a word file containing the genotype and allele frequencies of all the SNPs genotyped. [file 1471-2350-9-51-S1.doc]

**Table 3 - Genotype and Allele Frequencies**

| **CFH** | | Affected Siblings  (Index cases) | | Unaffected Siblings | |
| --- | --- | --- | --- | --- | --- |
|  |  | Frequency % | No. | Frequency % | No. |
| **rs800292** | **Genotype** |  |  |  |  |
|  | **GG** | 78.36 | 105 | 63.43 | 85 |
|  | **AG** | 18.66 | 25 | 30.60 | 41 |
|  | **AA** | 2.99 | 4 | 5.97 | 8 |
|  | **Total** |  | 134 |  | 134 |
|  |  |  |  |  |  |
|  | **Allele** |  |  |  |  |
|  | **G** | 87.69 | 235 | 78.73 | 211 |
|  | **A** | 12.31 | 33 | 21.27 | 57 |
|  | **Total** |  | 268 |  | 268 |

| **CFH** | | Affected Siblings  (Index cases) | | Unaffected Siblings | |
| --- | --- | --- | --- | --- | --- |
|  |  | Frequency % | No. | Frequency % | No. |
| **rs572515** | **Genotype** |  |  |  |  |
|  | **GG** | 20.90 | 28 | 35.34 | 47 |
|  | **AG** | 45.52 | 61 | 49.62 | 66 |
|  | **AA** | 33.58 | 45 | 15.04 | 20 |
|  | **Total** |  | 134 |  | 133 |
|  |  |  |  |  |  |
|  | **Allele** |  |  |  |  |
|  | **G** | 43.66 | 117 | 60.15 | 160 |
|  | **A** | 56.34 | 151 | 39.85 | 106 |
|  | **Total** |  | 268 |  | 266 |

| **CFH** | | Affected Siblings  (Index cases) | | Unaffected Siblings | |
| --- | --- | --- | --- | --- | --- |
|  |  | Frequency % | No. | Frequency % | No. |
| **rs7529589** | **Genotype** |  |  |  |  |
|  | **CC** | 23.31 | 31 | 32.09 | 43 |
|  | **TC** | 42.11 | 56 | 52.99 | 71 |
|  | **TT** | 34.59 | 46 | 14.93 | 20 |
|  | **Total** |  | 133 |  | 134 |
|  |  |  |  |  |  |
|  | **Allele** |  |  |  |  |
|  | **C** | 44.36 | 118 | 58.58 | 157 |
|  | **T** | 55.64 | 148 | 41.42 | 111 |
|  | **Total** |  | 266 |  | 268 |

| **CFH** | | Affected Siblings  (Index cases) | | Unaffected Siblings | |
| --- | --- | --- | --- | --- | --- |
|  |  | Frequency % | No. | Frequency % | No. |
| **rs12038333** | **Genotype** |  |  |  |  |
|  | **AA** | 21.64 | 29 | 32.09 | 43 |
|  | **GA** | 44.03 | 59 | 52.99 | 71 |
|  | **GG** | 34.33 | 46 | 14.93 | 20 |
|  | **Total** |  | 134 |  | 134 |
|  |  |  |  |  |  |
|  | **Allele** |  |  |  |  |
|  | **G** | 43.66 | 117 | 58.58 | 157 |
|  | **A** | 56.34 | 151 | 41.42 | 111 |
|  | **Total** |  | 268 |  | 268 |

| **CFH** | | Affected Siblings  (Index cases) | | Unaffected Siblings | |
| --- | --- | --- | --- | --- | --- |
|  |  | Frequency % | No. | Frequency % | No. |
| **rs203674** | **Genotype** |  |  |  |  |
|  | **TT** | 15.67 | 21 | 29.32 | 39 |
|  | **GT** | 43.28 | 58 | 48.87 | 65 |
|  | **GG** | 41.04 | 55 | 21.80 | 29 |
|  | **Total** |  | 134 |  | 133 |
|  |  |  |  |  |  |
|  | **Allele** |  |  |  |  |
|  | **T** | 37.31 | 100 | 53.76 | 143 |
|  | **A** | 62.69 | 168 | 46.24 | 123 |
|  | **Total** |  | 268 |  | 266 |

| **~CFH** | | Affected Siblings  (Index cases) | | Unaffected Siblings | |
| --- | --- | --- | --- | --- | --- |
|  |  | Frequency % | No. | Frequency % | No. |
| **rs10801575** | **Genotype** |  |  |  |  |
|  | **CC** | 65.35 | 83 | 54.03 | 67 |
|  | **TC** | 29.13 | 37 | 40.32 | 50 |
|  | **TT** | 5.51 | 7 | 5.65 | 7 |
|  | **Total** |  | 127 |  | 124 |
|  |  |  |  |  |  |
|  | **Allele** |  |  |  |  |
|  | **C** | 79.92 | 203 | 74.19 | 184 |
|  | **T** | 20.08 | 51 | 25.81 | 64 |
|  | **Total** |  | 254 |  | 248 |

| **CFHR4** | | Affected Siblings  (Index cases) | | Unaffected Siblings | |
| --- | --- | --- | --- | --- | --- |
|  |  | Frequency % | No. | Frequency % | No. |
| **rs1853883** | **Genotype** |  |  |  |  |
|  | **CC** | 15.04 | 20 | 26.12 | 35 |
|  | **GC** | 43.61 | 58 | 50.75 | 68 |
|  | **GG** | 41.35 | 55 | 23.13 | 31 |
|  | **Total** |  | 133 |  | 134 |
|  |  |  |  |  |  |
|  | **Allele** |  |  |  |  |
|  | **C** | 36.84 | 98 | 51.49 | 138 |
|  | **G** | 63.16 | 168 | 48.51 | 130 |
|  | **Total** |  | 266 |  | 268 |

| **CFHR2** | | Affected Siblings  (Index cases) | | Unaffected Siblings | |
| --- | --- | --- | --- | --- | --- |
|  |  | Frequency % | No. | Frequency % | No. |
| **rs3790414** | **Genotype** |  |  |  |  |
|  | **TT** | 73.13 | 98 | 59.70 | 80 |
|  | **AT** | 23.88 | 32 | 32.09 | 43 |
|  | **AA** | 2.99 | 4 | 8.21 | 11 |
|  | **Total** |  | 134 |  | 134 |
|  |  |  |  |  |  |
|  | **Allele** |  |  |  |  |
|  | **T** | 85.07 | 228 | 75.75 | 203 |
|  | **A** | 14.93 | 40 | 24.25 | 65 |
|  | **Total** |  | 268 |  | 268 |

| **CFHR5** | | Affected Siblings  (Index cases) | | Unaffected Siblings | |
| --- | --- | --- | --- | --- | --- |
|  |  | Frequency % | No. | Frequency % | No. |
| **rs1759016** | **Genotype** |  |  |  |  |
|  | **CC** | 55.64 | 74 | 43.28 | 58 |
|  | **TC** | 39.85 | 53 | 47.76 | 64 |
|  | **TT** | 4.51 | 6 | 8.96 | 12 |
|  | **Total** |  | 133 |  | 134 |
|  |  |  |  |  |  |
|  | **Allele** |  |  |  |  |
|  | **C** | 75.56 | 201 | 67.16 | 180 |
|  | **T** | 24.44 | 65 | 32.84 | 88 |
|  | **Total** |  | 266 |  | 268 |

| **CFHR5** | | Affected Siblings  (Index cases) | | Unaffected Siblings | |
| --- | --- | --- | --- | --- | --- |
|  |  | Frequency % | No. | Frequency % | No. |
| **rs10922152** | **Genotype** |  |  |  |  |
|  | **AA** | 41.35 | 55 | 27.07 | 36 |
|  | **TA** | 49.62 | 66 | 49.62 | 66 |
|  | **TT** | 9.02 | 12 | 23.31 | 31 |
|  | **Total** |  | 133 |  | 133 |
|  |  |  |  |  |  |
|  | **Allele** |  |  |  |  |
|  | **A** | 66.17 | 176 | 51.88 | 138 |
|  | **T** | 33.83 | 90 | 48.12 | 128 |
|  | **Total** |  | 266 |  | 266 |

| **CFHR5** | | Affected Siblings  (Index cases) | | Unaffected Siblings | |
| --- | --- | --- | --- | --- | --- |
|  |  | Frequency % | No. | Frequency % | No. |
| **rs10922153** | **Genotype** |  |  |  |  |
|  | **GG** | 42.11 | 56 | 25.78 | 33 |
|  | **TG** | 48.87 | 65 | 50.00 | 64 |
|  | **TT** | 9.02 | 12 | 24.22 | 31 |
|  | **Total** |  | 133 |  | 128 |
|  |  |  |  |  |  |
|  | **Allele** |  |  |  |  |
|  | **G** | 66.54 | 177 | 50.78 | 130 |
|  | **T** | 33.46 | 89 | 49.22 | 126 |
|  | **Total** |  | 266 |  | 256 |

|  | | Affected Siblings  (Index cases) | | Unaffected Siblings | |
| --- | --- | --- | --- | --- | --- |
|  |  | Frequency % | No. | Frequency % | No. |
| **rs6663083** | **Genotype** |  |  |  |  |
|  | **CC** | 40.60 | 54 | 26.12 | 35 |
|  | **TC** | 50.38 | 67 | 50.00 | 67 |
|  | **TT** | 9.02 | 12 | 23.88 | 32 |
|  | **Total** |  | 133 |  | 134 |
|  |  |  |  |  |  |
|  | **Allele** |  |  |  |  |
|  | **C** | 65.79 | 175 | 51.12 | 137 |
|  | **T** | 34.21 | 91 | 48.88 | 131 |
|  | **Total** |  | 266 |  | 268 |

| **F13B** | | Affected Siblings  (Index cases) | | Unaffected Siblings | |
| --- | --- | --- | --- | --- | --- |
|  |  | Frequency % | No. | Frequency % | No. |
| **rs2990510** | **Genotype** |  |  |  |  |
|  | **AA** | 41.04 | 55 | 47.76 | 64 |
|  | **CA** | 44.78 | 60 | 45.52 | 61 |
|  | **CC** | 14.18 | 19 | 6.72 | 9 |
|  | **Total** |  | 134 |  | 134 |
|  |  |  |  |  |  |
|  | **Allele** |  |  |  |  |
|  | **A** | 63.43 | 170 | 70.52 | 189 |
|  | **C** | 36.57 | 98 | 29.48 | 79 |
|  | **Total** |  | 268 |  | 268 |

| **F13B** | | Affected Siblings  (Index cases) | | Unaffected Siblings | |
| --- | --- | --- | --- | --- | --- |
|  |  | Frequency % | No. | Frequency % | No. |
| **rs6003** | **Genotype** |  |  |  |  |
|  | **AA** | 91.79 | 123 | 83.58 | 112 |
|  | **GA** | 8.21 | 11 | 15.67 | 21 |
|  | **GG** | 0.00 | 0 | 0.75 | 1 |
|  | **Total** |  | 134 |  | 134 |
|  |  |  |  |  |  |
|  | **Allele** |  |  |  |  |
|  | **A** | 95.90 | 257 | 91.42 | 245 |
|  | **G** | 4.10 | 11 | 8.58 | 23 |
|  | **Total** |  | 268 |  | 268 |

|  | | Affected Siblings  (Index cases) | | Unaffected Siblings | |
| --- | --- | --- | --- | --- | --- |
|  |  | Frequency % | No. | Frequency % | No. |
| **rs1412632** | **Genotype** |  |  |  |  |
|  | **TT** | 91.79 | 123 | 83.58 | 112 |
|  | **CT** | 8.21 | 11 | 15.67 | 21 |
|  | **CC** | 0.00 | 0 | 0.75 | 1 |
|  | **Total** |  | 134 |  | 134 |
|  |  |  |  |  |  |
|  | **Allele** |  |  |  |  |
|  | **T** | 95.90 | 257 | 91.42 | 245 |
|  | **C** | 4.10 | 11 | 8.58 | 23 |
|  | **Total** |  | 268 |  | 268 |

|  | | Affected Siblings  (Index cases) | | Unaffected Siblings | |
| --- | --- | --- | --- | --- | --- |
|  |  | Frequency % | No. | Frequency % | No. |
| **rs1412631** | **Genotype** |  |  |  |  |
|  | **GG** | 92.54 | 124 | 84.33 | 113 |
|  | **AG** | 7.46 | 10 | 14.93 | 20 |
|  | **AA** | 0.00 | 0 | 0.75 | 1 |
|  | **Total** |  | 134 |  | 134 |
|  |  |  |  |  |  |
|  | **Allele** |  |  |  |  |
|  | **G** | 96.27 | 258 | 91.79 | 246 |
|  | **A** | 3.73 | 10 | 8.21 | 22 |
|  | **Total** |  | 268 |  | 268 |

| **ASPM** | | Affected Siblings  (Index cases) | | Unaffected Siblings | |
| --- | --- | --- | --- | --- | --- |
|  |  | Frequency % | No. | Frequency % | No. |
| **rs12677** | **Genotype** |  |  |  |  |
|  | **TT** | 92.54 | 124 | 84.33 | 113 |
|  | **CT** | 7.46 | 10 | 14.93 | 20 |
|  | **CC** | 0.00 | 0 | 0.75 | 1 |
|  | **Total** |  | 134 |  | 134 |
|  |  |  |  |  |  |
|  | **Allele** |  |  |  |  |
|  | **T** | 96.27 | 258 | 91.79 | 246 |
|  | **C** | 3.73 | 10 | 8.21 | 22 |
|  | **Total** |  | 268 |  | 268 |

| **ASPM** | | Affected Siblings  (Index cases) | | Unaffected Siblings | |
| --- | --- | --- | --- | --- | --- |
|  |  | Frequency % | No. | Frequency % | No. |
| **rs4915337** | **Genotype** |  |  |  |  |
|  | **AA** | 91.79 | 123 | 85.71 | 114 |
|  | **TA** | 8.21 | 11 | 13.53 | 18 |
|  | **TT** | 0.00 | 0 | 0.75 | 1 |
|  | **Total** |  | 134 |  | 133 |
|  |  |  |  |  |  |
|  | **Allele** |  |  |  |  |
|  | **A** | 95.90 | 257 | 92.48 | 246 |
|  | **T** | 4.10 | 11 | 7.52 | 20 |
|  | **Total** |  | 268 |  | 266 |

| **ASPM** | | Affected Siblings  (Index cases) | | Unaffected Siblings | |
| --- | --- | --- | --- | --- | --- |
|  |  | Frequency % | No. | Frequency % | No. |
| **rs1888991** | **Genotype** |  |  |  |  |
|  | **CC** | 91.79 | 123 | 84.21 | 112 |
|  | **TC** | 8.21 | 11 | 15.04 | 20 |
|  | **TT** | 0.00 | 0 | 0.75 | 1 |
|  | **Total** |  | 134 |  | 133 |
|  |  |  |  |  |  |
|  | **Allele** |  |  |  |  |
|  | **C** | 95.90 | 257 | 91.73 | 244 |
|  | **T** | 4.10 | 11 | 8.27 | 22 |
|  | **Total** |  | 268 |  | 266 |

| **ZBTB41** | | Affected Siblings  (Index cases) | | Unaffected Siblings | |
| --- | --- | --- | --- | --- | --- |
|  |  | Frequency % | No. | Frequency % | No. |
| **rs6656448** | **Genotype** |  |  |  |  |
|  | **AA** | 91.04 | 122 | 82.84 | 111 |
|  | **GA** | 8.96 | 12 | 16.42 | 22 |
|  | **GG** | 0.00 | 0 | 0.75 | 1 |
|  | **Total** |  | 134 |  | 134 |
|  |  |  |  |  |  |
|  | **Allele** |  |  |  |  |
|  | **A** | 95.52 | 256 | 91.04 | 244 |
|  | **G** | 4.48 | 12 | 8.96 | 24 |
|  | **Total** |  | 268 |  | 268 |

| **MAP2** | | Affected Siblings  (Index cases) | | Unaffected Siblings | |
| --- | --- | --- | --- | --- | --- |
|  |  | Frequency % | No. | Frequency % | No. |
| **rs9288410** | **Genotype** |  |  |  |  |
|  | **GG** | 77.61 | 104 | 80.30 | 106 |
|  | **AG** | 20.15 | 27 | 18.94 | 25 |
|  | **AA** | 2.24 | 3 | 0.76 | 1 |
|  | **Total** |  | 134 |  | 132 |
|  |  |  |  |  |  |
|  | **Allele** |  |  |  |  |
|  | **G** | 87.69 | 235 | 89.77 | 237 |
|  | **A** | 12.31 | 33 | 10.23 | 27 |
|  | **Total** |  | 268 |  | 264 |

| **ITPR1** | | Affected Siblings  (Index cases) | | Unaffected Siblings | |
| --- | --- | --- | --- | --- | --- |
|  |  | Frequency % | No. | Frequency % | No. |
| **rs304039** | **Genotype** |  |  |  |  |
|  | **TT** | 36.57 | 49 | 34.33 | 46 |
|  | **GT** | 41.04 | 55 | 48.51 | 65 |
|  | **GG** | 22.39 | 30 | 17.16 | 23 |
|  | **Total** |  | 134 |  | 134 |
|  |  |  |  |  |  |
|  | **Allele** |  |  |  |  |
|  | **T** | 57.09 | 153 | 58.58 | 157 |
|  | **G** | 42.91 | 115 | 41.42 | 111 |
|  | **Total** |  | 268 |  | 268 |

| **ITPR1** | | Affected Siblings  (Index cases) | | Unaffected Siblings | |
| --- | --- | --- | --- | --- | --- |
|  |  | Frequency % | No. | Frequency % | No. |
| **rs304041** | **Genotype** |  |  |  |  |
|  | **TT** | 37.31 | 50 | 38.06 | 51 |
|  | **GT** | 40.30 | 54 | 44.78 | 60 |
|  | **GG** | 22.39 | 30 | 17.16 | 23 |
|  | **Total** |  | 134 |  | 134 |
|  |  |  |  |  |  |
|  | **Allele** |  |  |  |  |
|  | **T** | 57.46 | 154 | 60.45 | 162 |
|  | **G** | 42.54 | 114 | 39.55 | 106 |
|  | **Total** |  | 268 |  | 268 |

| **ITPR1** | | Affected Siblings  (Index cases) | | Unaffected Siblings | |
| --- | --- | --- | --- | --- | --- |
|  |  | Frequency % | No. | Frequency % | No. |
| **rs1038639** | **Genotype** |  |  |  |  |
|  | **GG** | 29.85 | 40 | 26.87 | 36 |
|  | **TG** | 45.52 | 61 | 50.00 | 67 |
|  | **TT** | 24.63 | 33 | 23.13 | 31 |
|  | **Total** |  | 134 |  | 134 |
|  |  |  |  |  |  |
|  | **Allele** |  |  |  |  |
|  | **G** | 52.61 | 141 | 51.87 | 139 |
|  | **T** | 47.39 | 127 | 48.13 | 129 |
|  | **Total** |  | 268 |  | 268 |

|  | | Affected Siblings  (Index cases) | | Unaffected Siblings | |
| --- | --- | --- | --- | --- | --- |
|  |  | Frequency % | No. | Frequency % | No. |
| **rs1447338** | **Genotype** |  |  |  |  |
|  | **AA** | 53.38 | 71 | 57.14 | 76 |
|  | **GA** | 37.59 | 50 | 34.59 | 46 |
|  | **GG** | 9.02 | 12 | 8.27 | 11 |
|  | **Total** |  | 133 |  | 133 |
|  |  |  |  |  |  |
|  | **Allele** |  |  |  |  |
|  | **A** | 72.18 | 192 | 74.44 | 198 |
|  | **G** | 27.82 | 74 | 25.56 | 68 |
|  | **Total** |  | 266 |  | 266 |

| **ADD3** | | Affected Siblings  (Index cases) | | Unaffected Siblings | |
| --- | --- | --- | --- | --- | --- |
|  |  | Frequency % | No. | Frequency % | No. |
| **rs7090030** | **Genotype** |  |  |  |  |
|  | **TT** | 99.25 | 133 | 98.51 | 132 |
|  | **GT** | 0.75 | 1 | 1.49 | 2 |
|  | **GG** | 0.00 | 0 | 0.00 | 0 |
|  | **Total** |  | 134 |  | 134 |
|  |  |  |  |  |  |
|  | **Allele** |  |  |  |  |
|  | **T** | 99.63 | 267 | 99.25 | 266 |
|  | **G** | 0.37 | 1 | 0.75 | 2 |
|  | **Total** |  | 268 |  | 268 |

| **ADD3** | | Affected Siblings  (Index cases) | | Unaffected Siblings | |
| --- | --- | --- | --- | --- | --- |
|  |  | Frequency % | No. | Frequency % | No. |
| **rs11194995** | **Genotype** |  |  |  |  |
|  | **CC** | 99.25 | 133 | 98.51 | 132 |
|  | **TC** | 0.75 | 1 | 1.49 | 2 |
|  | **TT** | 0.00 | 0 | 0.00 | 0 |
|  | **Total** |  | 134 |  | 134 |
|  |  |  |  |  |  |
|  | **Allele** |  |  |  |  |
|  | **C** | 99.63 | 267 | 99.25 | 266 |
|  | **T** | 0.37 | 1 | 0.75 | 2 |
|  | **Total** |  | 268 |  | 268 |

| **ADD3** | | Affected Siblings  (Index cases) | | Unaffected Siblings | |
| --- | --- | --- | --- | --- | --- |
|  |  | Frequency % | No. | Frequency % | No. |
| **rs11194996** | **Genotype** |  |  |  |  |
|  | **CC** | 98.51 | 132 | 99.25 | 133 |
|  | **GC** | 1.49 | 2 | 0.75 | 1 |
|  | **GG** | 0.00 | 0 | 0.00 | 0 |
|  | **Total** |  | 134 |  | 134 |
|  |  |  |  |  |  |
|  | **Allele** |  |  |  |  |
|  | **C** | 99.25 | 266 | 99.63 | 267 |
|  | **G** | 0.75 | 2 | 0.37 | 1 |
|  | **Total** |  | 268 |  | 268 |

| **ADD3** | | Affected Siblings  (Index cases) | | Unaffected Siblings | |
| --- | --- | --- | --- | --- | --- |
|  |  | Frequency % | No. | Frequency % | No. |
| **rs11195001** | **Genotype** |  |  |  |  |
|  | **TT** | 99.25 | 132 | 98.50 | 131 |
|  | **CT** | 0.75 | 1 | 1.50 | 2 |
|  | **CC** | 0.00 | 0 | 0.00 | 0 |
|  | **Total** |  | 133 |  | 133 |
|  |  |  |  |  |  |
|  | **Allele** |  |  |  |  |
|  | **T** | 99.62 | 265 | 99.25 | 264 |
|  | **C** | 0.38 | 1 | 0.75 | 2 |
|  | **Total** |  | 266 |  | 266 |

|  | | Affected Siblings  (Index cases) | | Unaffected Siblings | |
| --- | --- | --- | --- | --- | --- |
|  |  | Frequency % | No. | Frequency % | No. |
| **rs2014307** | **Genotype** |  |  |  |  |
|  | **GG** | 56.72 | 76 | 36.09 | 48 |
|  | **TG** | 32.09 | 43 | 43.61 | 58 |
|  | **TT** | 11.19 | 15 | 20.30 | 27 |
|  | **Total** |  | 134 |  | 133 |
|  |  |  |  |  |  |
|  | **Allele** |  |  |  |  |
|  | **G** | 72.76 | 195 | 57.89 | 154 |
|  | **T** | 27.24 | 73 | 42.11 | 112 |
|  | **Total** |  | 268 |  | 266 |

| **GPR152** | | Affected Siblings  (Index cases) | | Unaffected Siblings | |
| --- | --- | --- | --- | --- | --- |
|  |  | Frequency % | No. | Frequency % | No. |
| **rs949252** | **Genotype** |  |  |  |  |
|  | **TT** | 98.51 | 132 | 99.25 | 133 |
|  | **CT** | 1.49 | 2 | 0.00 | 0 |
|  | **CC** | 0.00 | 0 | 0.75 | 1 |
|  | **Total** |  | 134 |  | 134 |
|  |  |  |  |  |  |
|  | **Allele** |  |  |  |  |
|  | **T** | 99.25 | 266 | 99.25 | 266 |
|  | **C** | 0.75 | 2 | 0.75 | 2 |
|  | **Total** |  | 268 |  | 268 |

| **TMEM134** | | Affected Siblings  (Index cases) | | Unaffected Siblings | |
| --- | --- | --- | --- | --- | --- |
|  |  | Frequency % | No. | Frequency % | No. |
| **rs7124630** | **Genotype** |  |  |  |  |
|  | **TT** | 99.25 | 133 | 98.51 | 132 |
|  | **CT** | 0.75 | 1 | 0.75 | 1 |
|  | **CC** | 0.00 | 0 | 0.75 | 1 |
|  | **Total** |  | 134 |  | 134 |
|  |  |  |  |  |  |
|  | **Allele** |  |  |  |  |
|  | **T** | 99.63 | 267 | 98.88 | 265 |
|  | **C** | 0.37 | 1 | 1.12 | 3 |
|  | **Total** |  | 268 |  | 268 |

|  | | Affected Siblings  (Index cases) | | Unaffected Siblings | |
| --- | --- | --- | --- | --- | --- |
|  |  | Frequency % | No. | Frequency % | No. |
| **rs11575221** | **Genotype** |  |  |  |  |
|  | **AA** | 98.51 | 132 | 99.25 | 133 |
|  | **CA** | 1.49 | 2 | 0.75 | 1 |
|  | **CC** | 0.00 | 0 | 0.00 | 0 |
|  | **Total** |  | 134 |  | 134 |
|  |  |  |  |  |  |
|  | **Allele** |  |  |  |  |
|  | **A** | 99.25 | 266 | 99.63 | 267 |
|  | **C** | 0.75 | 2 | 0.37 | 1 |
|  | **Total** |  | 268 |  | 268 |

**Abbreviations:** No., number.
